# Factors Influencing Self-Management among Non-Dialysis Chronic Kidney Disease Patients

**DOI:** 10.3390/healthcare10030436

**Published:** 2022-02-25

**Authors:** Hayfa Almutary, Nahla Tayyib

**Affiliations:** 1 Medical Surgical Nursing Department, Faculty of Nursing, King Abdulaziz University, Jeddah 22246, Saudi Arabia; 2 Nursing Practices Department, Faculty of Nursing, Umm Al-Qura University, Makkah 21955, Saudi Arabia; natayyib@uqu.edu.sa; 3 Deanship of Scientific Research, Umm Al-Qura University, Makkah 21955, Saudi Arabia

**Keywords:** chronic kidney disease, kidney disease knowledge, nursing care, non-dialysis, self-management

## Abstract

Aim: The new trend in the management of chronic kidney disease (CKD) is based on the adoption of self-management approaches. However, there is a paucity of research assessing the level of self-management behavior among non-dialysis patients. The aim of the study is to assess the association between self-management behaviors and the level of disease-specific knowledge among non-dialysis CKD patients. In addition, this study aimed to assess the predictors of self-management among non-CKD patients. Methods: A convenience sample of 203 non-dialysis patients with stage 3–5 CKD was surveyed from the nephrology clinics in Saudi Arabia. Descriptive statistics and linear regression were used to analyze the data. Results: The mean level of knowledge and self-management was 17.9 ± 3.2 and 76.9 ± 13.3, respectively. The results of the multiple regression of self-management showed that knowledge was independently associated with self-management (*r* = 0.51, **, *p* < 0.001). Conclusions: This study demonstrated that knowledge and self-management were associated with each other in non-dialysis patients. More efforts are needed to track and enhance the knowledge levels in patients with CKD. Future research should focus on the effectiveness of educational programs of self-management behavior.

## 1. Introduction

Chronic kidney disease (CKD) is becoming a public health issue with a global prevalence of 13.4% in stages 1–5 and 10.6% in stages 3–5 [1]. The actual prevalence of CKD is expected to be higher than the reported values because it is often diagnosed by accident. CKD is a silent disease that can progress to end-stage disease and can lead to serious complications without any obvious symptoms. The prevalence of CKD is projected to increase by 16.7% among adults by 2030 [2]. In addition, global epidemiological data show that 1.2 million individuals died from CKD in 2017 and that this mortality rate increased by 41.5% from 1990 to 2017 [3]. These high prevalence and mortality rates are probably due to insufficient management of the disease and population aging [3].

CKD is a complex disease with multidimensional effects on people’s lives. With disease progression, people with CKD often experience multiple symptoms and develop cardiovascular complications and other comorbidities, which require continuous monitoring and care [4]. Caring for these patients involves preservation of physical and psychological functioning, delaying disease progression, and preventing potential complications [5]. An extensive body of literature suggests that a part of the caring responsibility should be conducted by patients as self-management [6,7]. In addition, a new trend in the management of CKD involves a patient-centered approach focused on self-management. In this approach, CKD management focuses on the person’s self-management performance, which generally aims to simplify a person’s capacity to create lifestyle modifications and manage symptoms, medications, and the physical and psychosocial side effects of CKD and its related comorbidities. Patients with CKD are assessed on five dimensions: communication, partnership in care, self-care activities, self-integration, and adherence to recommended treatment. It includes treatment regimens, early recognition of warning signs (e.g., blood pressure and glucose monitoring), and lifestyle changes such as diet recommendations and physical activity [8].

Self-management can improve overall healthcare outcomes among non-dialysis CKD patients and may delay the progression of the disease and reduce the occurrence of morbidities [4,7,9]. In addition, there is some evidence showing that patients with stage 1–5 CKD who received self-management programs have lower hospitalization rates [10] and better medication adherence than others on routine care [11]. A recent study has shown that there is a strong relationship between self-management and knowledge in the early stages of CKD [5]. Gaining sufficient knowledge could enhance self-management behavior. Therefore, people with CKD need to sustain self-management behaviors with the willingness to participate in the optimization of their daily life demands, which requires sufficient knowledge about their disease and its management.

Self-management behavior requires concentrating on disease needs, which requires sufficient knowledge, good competencies, and confidence in medical prospects [12,13]. People with CKD should have sufficient knowledge about their condition and understand basic health information to manage their disease [14]. Multiple studies have shown an association between an individual’s understanding of CKD and clinical outcomes [15,16]. For example, people with CKD who understand blood pressure (BP) monitoring goals show ameliorated BP, and those who are aware of dialysis often have permanent arteriovenous access [17,18]. Furthermore, 23% of people with CKD have a low level of kidney disease knowledge, which has been associated with worsening of clinical outcomes [19,20].

Nurses play a key role in the development of the CKD patients’ self-management skills. However, monitoring remains a challenge because the patients must be highly engaged, but many patients’ level of commitment remains inadequate [6]. In addition, people with CKD face several challenges, including insufficient information concerning guidelines and recommendations for managing and controlling CKD [21].

Limited data are available worldwide regarding knowledge and self-management among non-dialysis CKD patients. Several studies have shown that knowledge levels among non-dialysis patients are low to moderate [5,22,23], whereas others have shown a low level of self-management among patients with CKD [5,24,25]. Of these, studies addressing the correlation between these two concepts are limited. Chuang et al. (2020) showed that disease knowledge and self-management are strongly associated with the early stages of CKD [5]. Moreover, findings from a qualitative study showed that there is a window for improving CKD self-management by enhancing knowledge [26]. In Saudi Arabia, no data exist regarding self-management behaviors and the level of knowledge among non-dialysis CKD patients. Understanding this relationship among non-dialysis patients will help nurses and other healthcare professionals to implement appropriate interventions that enhance self-management behaviors in this population.

## 2. Purpose of the Study

This study aimed to assess self-management behaviors and CKD disease knowledge levels among non-dialysis CKD patients and to examine the associations between these two factors. It also aimed to assess the predictors of self-management among non-CKD patients.

## 3. Methodology

### 3.1. Design

A correlational cross-sectional design was used in this study to assess self-management behaviors and the level of kidney disease knowledge among adults with CKD.

### 3.2. Participants and Setting

A convenience sample of patients with CKD was recruited from different nephrology clinics in two hospitals in the western region of the Kingdom of Saudi Arabia from October 2019 to January 2020. A total of 203 patients with CKD met the following inclusion criteria: age ≥ 18 years, diagnosis of stages 3–5 of CKD, ability to understand Arabic, and willingness to participate in the study. However, we excluded patients who had a kidney transplant or were commencing dialysis therapy and those who were cognitively impaired.

The sample size was calculated using G* Power 3.1 software by assuming 95% power (1-beta = 0.95), a type 1 error rate (alpha) of 0.05 (two-tailed), and a large effect size (Cohen’s d = 0.1). These criteria are recommended in observational studies to ensure greater power and higher representation of the study population [27]. Therefore, a minimum of 133 participants was required.

### 3.3. Measures

Demographic and clinical data that could be associated with self-management behaviors were collected. These included age, sex, marital status, education level, occupation status, time since CKD diagnosis, body mass index (BMI), and BP levels. Although obesity and uncontrolled BP can be predictors of low self-management, these factors have been less frequently evaluated; thus, we included them in our study.

CKD-related knowledge was measured using the Kidney Disease Knowledge Survey (KiKS) which has 28 questions; every correct answer is assigned one point. This instrument mainly focuses on determining the level of patient knowledge about specific areas, such as kidney function, treatment options, signs and symptoms, and targeted BP. The content of this instrument is aligned with self-management behavior, which reflects changes in behavior. The total score can be obtained by summing the correct responses, which range from 0 to 28, with 28 indicating the highest level of knowledge [28]. This instrument is valid and reliable and has been used in non-dialysis CKD patients [25,28,29].

CKD self-management behavior was evaluated using the Chronic Kidney Disease Self-Management-29 (CKD-SM-29) instrument. It has 29 items covering four subscales: self-integration (11 items), problem-solving (9 items), seeking social support (5 items), and adherence to recommended regimen (4 items). Each item is measured on a scale of 1 (never) to 4 (always), and the total score for this instrument was calculated by summing the score of each item, with the final score ranging from 29 to 116. The highest total score indicates more effective CKD self-management behavior [25,30]. This instrument was developed specifically for non-dialysis CKD patients and has been demonstrated to be valid and reliable [30].

Both instruments were translated into Arabic using the backward translation technique and validated in patients with CKD [31]. The internal consistency of the Arabic version of the KiKS and CKD-SM-29 was 0.75 and 0.91, respectively. The intra-class correlation coefficient to determine the inter-rater reliability for the CKD-SM-29 was 0.90, and the corresponding value for the Arabic version of the KiKS was 0.87. Convergent and discriminative validity were also demonstrated for the translated instruments [31].

### 3.4. Data Collection

To confirm the disease stage, the last updated serum creatinine level and urine protein results were checked and classified according to the National Kidney Foundation Kidney Disease Outcomes Quality Initiative guidelines. Eligible participants were selected by the assistant to the head nurse who reviewed all booked appointments in nephrology clinics. Eligible participants were approached in the waiting areas of nephrology clinics. The research was then explained verbally and in writing; if a participant was willing to take part in this study, the participant completed the consent form and was provided with study materials. One of the authors of this study was responsible for data collection, which was performed using a self-reported questionnaire. Other clinical data were obtained from the hospital files. In the case of illiterate patients, the same researcher helped in filling out the study questionnaires by loudly and clearly reading the items exactly as they were written in the questionnaires without any further clarification. Additionally, the researcher has ensured that the participants have adequate time to answer the questionnaires. Using this technique, participants will obtain similar instructions, which minimizes latent bias that could affect their responses [32]. This study was approved by the research ethics board of the hospital (reference no. HA.-02-J.008).

### 3.5. Data Analysis

Data were entered into the statistical software IBM SPSS version 25. Few missing data were found in the overall instruments (<2%) and were missing completely at random. Therefore, missing data were substituted by mean values. The data were assessed for linearity, outliers, normality, and collinearity. Frequencies and percentages were used to describe the distribution of patients’ demographic and clinical data. The knowledge and self-management questions were assessed and scored. To calculate the total score of the KiKS, one (+1) mark was given for each correct response, zero (0) was assigned for each incorrect response, and the scores for the correct answers were summed. The Pearson correlation coefficient was used to examine the relationships between knowledge and self-management.

A multiple regression analysis was performed to assess the predictors of self-management. To build the model, we ran an enter approach with the predictor variables. The predictors were selected according to literature in relation to demographic and clinical variables, in addition to the statistical significance of the bivariate relationships. The Pearson correlation test was used to assess the associations between continuous variables. Therefore, the final model included age, BMI, educational status, time since aware CKD diagnosis, and knowledge. Statistical significance was set at *p* < 0.05.

## 4. Results

### 4.1. Demographic and Clinical Characteristics

The characteristics of the 203 participants are displayed in Table 1. The participant population had a mean age of 47.3 ± 12.1 years. Half of the participants were male (50.2%), and 75.9% were married. Most of them went to high school or lower (57.1%), with 21.7% having a bachelor’s degree. Only 18.7% of participants were currently working. The mean BMI was 28.8 ± 6.6 kg/m^2^, which falls into the overweight category. More than one-third of the participants (38.4%) had been aware of their diagnosis of CKD for less than four years.

### 4.2. Descriptive Statistics for the CKD-SM-29 and KiKS

The maximum possible total score on the KiKS was 28. In this study, the scores ranged from 6 to 25, with a mean of 17.9 ± 3.2. For the CKD-SM-29, the possible minimum and maximum scores were 29 and 116, respectively. In this study, the scores ranged from 43 to 116, with a mean of 76.9 ± 13.3 (see Table 2). Furthermore, Table 2 presents an overview score of the four subscales of self-management behaviors in the CKD population: (1) self-integration, (2) problem-solving, (3) seeking social support, and (4) adherence to the recommended regimen. However, adherence to the recommended regimen subscale had the lowest score in comparison to the other subscales (M = 9.6 ± 2.8).

### 4.3. Predictors of CKD Self-Management

The findings of multivariate analysis for the key variables show significant correlation between self-management behavior and knowledge, age, BMI, educational status, and time aware of CKD (*r* = 0.514, *p* < 0.001; *r* = −0.165, *p* = 0.029; *r* = −0.149, *p* = 0.034; *r* = 0.15, *p* = 0.043; *r* = −0.16, *p* = 0.023, respectively).

Multiple regression analysis (Table 3) was performed to explore the association between demographic and clinical characteristics, kidney disease knowledge, and self-management behavior. The results showed that kidney disease knowledge, age, and BMI were independently associated with self-management behavior scores. Based on the standardized coefficients, kidney disease knowledge was the strongest influencing factor, followed by age. The model explained 29% of the variation in the total self-management score.

## 5. Discussion

This study provides primary evidence of self-management behavior among non-dialysis CKD patients. Worldwide, data on this specific population are limited. Moreover, this study is the first to assess self-management behavior in relation to the level of knowledge among non-dialysis CKD patients in Saudi Arabia. Overall, self-management levels and knowledge were considered to be low among patients with CKD. The mean score for self-management was 76.9 ± 13.3, which is lower than that of previously reported levels in international studies [24,25]. In addition, the mean knowledge score in our study was 17.9 ± 3.2. This finding is also lower than that reported in previous international studies [22,23]. The slight variation may be due to the difference in disease stage; our patients were between stages 3 and 5 only. In addition, only 21.7% of our patients had a high education level, which could explain the poor knowledge level in our sample. Other factors that may contribute to the poor knowledge level of non-dialysis patients are levels of communication between healthcare professionals and patients, which could be the result of the number of clients per clinic, insufficient information, and handover of patient care between caregivers. Clients’ satisfaction with communication has been considered to be one of the factors influencing perceived knowledge in the CKD population [23,28]. Moreover, effective communication has a positive influence on compliance with treatment regimens for chronic illnesses [33]. Moreover, little training that may be offered to patients could be another factor contributing to low knowledge among non-dialysis patients.

Our study also demonstrated a strong association between knowledge and self-management among non-dialysis patients; a higher level of knowledge is associated with better self-management behaviors. This result is consistent with a more recent study that reported relationships among higher CKD knowledge levels, positive attitudes, and self-management [24].

In this study, knowledge was the strongest predictor of self-management after controlling for age, education level, BMI, and CKD diagnosis. This result aligns with several previous studies that described patients with kidney disease and other chronic diseases [34,35]. Previous studies of people with CKD have shown that better disease-related knowledge is associated with better self-management behavior, which was demonstrated by improved BP control [18,29]. Therefore, people who are more knowledgeable about their disease are likely to be more confident in managing the disease. This result suggests that empowering knowledge through educational programs for these patients is needed to achieve a positive outcome. Interestingly, education level was not found as a predictor of self-management in this study. It appears that health literacy and specific disease knowledge need to be assessed for all patients, regardless their educational level.

Several studies have shown that educating patients is associated with longer survival rates and delayed initiation of dialysis therapy [36,37]. This suggests that there are patients ready to engage in better management, and the earlier the stage of the disease, the more crucial and beneficial it is to educate patients and enlighten their acquaintances. The main reason for this is that non-dialysis CKD patients have a greater chance of sustaining effective outcomes from educational interventions [38]. Nurses should assess individuals’ level of knowledge about CKD using standardized measures and record this baseline information as one of the main goals embedded in the treatment plan, which is directed towards active self-management [39]. However, time constraints in clinics have been recognized as an important obstacle to providing non-dialysis CKD patients with sufficient knowledge of CKD [40,41]. Applying such innovative approaches to CKD education—such as SMS, shared decision-making, and the use of digital media—may enhance knowledge [41].

Age is another important predictor of self-management among non-dialysis patients. We found that older age was significantly associated with lower self-management behaviors in CKD, which is understandable given that elderly people have a low capacity for engagement [42]. More attention is needed for these patient groups. Further efforts to design appropriate interventions to enhance self-management skills of older people with CKD, which could involve family members, may be beneficial in helping them better deal with kidney disease. Furthermore, this study found a converse association between BMI and self-management behaviors in non-dialysis patients, which indicates that a higher BMI is a predictor of lower self-management behaviors. This is probably because the clients are more confident and aware, and adhere to the treatment plan, which includes eating behaviors and physical activity on daily basis, the most challenging factors in controlling patient weight.

To date, evidence on self-management for patients with earlier stages of kidney disease is still insufficient. To improve active self-management behaviors and facilitate decision-making for non-dialysis CKD patients at different stages (3–5), specific knowledge related to kidney disease should be assessed and improved. This is the first Saudi Arabian study to highlight such a topic with significant results.

Although this study has significant implications for practice and future research, it also has some limitations. Predictive variables such as social support and health literacy, which could be associated with self-management, need to be addressed in future studies. The cross-sectional design causes a temporality problem in the proven associations. In addition, the generalizability of the findings may be limited because of the narrow study settings, which included only the western region of Saudi Arabia. In addition, some sources of bias that could occur with a cross-sectional design and self-reported survey should be acknowledged, such as selection and recall biases.

## 6. Conclusions

This is the first study to assess self-management behavior and level of disease-specific knowledge in non-dialysis CKD patients and to examine the associations between them in Saudi Arabia. The findings of this study will contribute to the growing body of literature. In addition, a better understanding of the relationships between knowledge and self-management behaviors among non-dialysis patients will help nurses implement appropriate interventions to enhance self-management in this population.

The findings of this study underscore the importance of enhancing disease-specific knowledge about CKD in order to improve self-management behaviors during the early stages of the disease. Nurses and other healthcare professionals need to assess disease knowledge among CKD patients and make appropriate education plans to enhance their knowledge. However, it seems that non-dialysis CKD patients in stages 3–5 are not provided with sufficient knowledge at clinics, which may be due to the time constraints of the healthcare professionals. However, when nurses prioritize educating these populations, a significant positive outcome may result. Involving patients as decision makers during the formation of the treatment plan may enhance adherence to the recommended therapy regimen and therefore lead to better self-management. Considering the severe time constraints in clinics, policymakers need to facilitate the application of such innovative approaches—such as SMS, shared decision-making, and the use of digital media—to patient education. Therefore, improved CKD knowledge is crucial for improving self-management in CKD, especially in the early stages, thereby promoting human health and preventing the progression of CKD. Future research should focus on evaluating the effectiveness of educational programs on self-management behavior.

## Figures and Tables

**Table 1:** Characteristics of the study population, *n* = 203.

Variable	*n*	%	Mean	SD	Range
Age			47.3	12.1	18–68
Gender					
Male	102	50.2			
Female	101	49.8			
Marital status					
Unmarried	49	24.1			
Married	154	75.9			
Education level					
Illiterate	41	20.2			
High School or less	116	57.1			
Bachelor’s degree	44	21.7			
Postgraduate	2	1.0			
Occupational status					
Unemployed	44	21.7			
Household	72	35.5			
Employee	38	18.7			
Retired	49	24.1			
Time since diagnosis					
0–12 months (less than a year)	64	31.5			
1–4 years	78	38.4			
5–10 years	31	15.3			
More than 10 years	30	14.8			
Body mass index			28.8	6.6	16–36
Blood pressure (mmHg)					
Systolic			134.4	16.4	99–170
Diastolic			80.6	10.9	50–110

SD: standard deviation.

**Table 2:** Descriptive statistics for the CKD-SM-29 and KiKS.

Subscale	Potential Range	Actual Range	Mean	SD
Self-integration	11–44	16–44	30.2	5.6
Problem solving	9–36	14–36	24.4	4.8
Seeking social support	5–20	5–20	12.8	2.8
Adherence to recommended regimen	4–16	4–16	9.6	2.8
Total score for CKD-SM-29	29–116	43–116	76.9	13.3
Total score for KiKS	0–28	6–25	17.9	3.2

KiKS: Kidney Disease Knowledge Survey; SD: standard deviation; CKD-SM-29: Chronic Kidney Disease Self-Management-29; Potential range: the minimum and maximum expected range of the scales; Actual range: range of each scale that we got in this study.

**Table 3:** Multiple regression analysis for variables predicting self-management.

General CKD Self-Management	Reference	(F = 18.54, *p* ˂ 0.001, Adjusted R^2^ = 0.29)		*p*-Value *
		*β*	95% CI	
Constant	-	0	30.77, 57.63	<0.0001
Knowledge	-	0.513	1.45, 2.29	<0.0001
Educational Status	High School or less	0.023	−1.92, 2.84	0.705
Age	-	−0.183	−0.33, −0.06	0.004
BMI	-	−0.159	−0.076, −0.56	0.010
Time aware of CKD	0–12 months (less than a year)	−0.050	−2.19, 0.88	0.405

*β* = Standardized regression coefficient. * A *p*-value < 0.05 was considered to be statistically significant.

## Data Availability

The data presented are included in this study; the corresponding author on request may provide additional data.
